# An Under-the-Table Leg-Movement Apparatus and Changes in Energy Expenditure

**DOI:** 10.3389/fphys.2017.00318

**Published:** 2017-05-18

**Authors:** Gabriel A. Koepp, Graham Moore, James A. Levine

**Affiliations:** ^1^Obesity Solutions, Mayo ClinicScottsdale, AZ, United States; ^2^Obesity Solutions, Arizona State UniversityTempe, AZ, United States

**Keywords:** energy expenditure, fidget, non-exercise activity thermogenesis, sedentary behavior, sitting disease

## Abstract

**Introduction:** Deskwork contributes substantially to sedentariness. Here, we evaluated an under-the-table apparatus that was designed to promote leg movement (fidgeting) while seated. Our hypothesis was that the under-the-table apparatus would increase energy expenditure.

**Methods:** We measured energy expenditure and heart rate in 26 people while they sat and worked using a standard chair, walked on a treadmill, and sat and worked using an under-the-desk apparatus that encouraged leg movement.

**Results:** Energy expenditure increased significantly while using the under-the-table apparatus when compared to the standard office chair (standard chair, 81 ± 18 kcal/h; under-the-table apparatus, 96 ± 23 kcal/h) (*P* < 0.001); representing an 18 ± 16% increase. The changes in energy expenditure were not as great as walking (1 mph, 168 ± 46 kcal/h, *P* < 0.001; 2 mph, 205 ± 51 kcal/, *P* < 0.001), representing 107 ± 37% and 155 ± 48% increases over baseline, respectively.

**Conclusions:** An under-the-table apparatus that promotes leg movement can increase energy expenditure by approximately 20%. Dynamic sitting is promoted by this apparatus and may be among a lexicon of options to help people move more while seated at work.

## Introduction

Sitting excessively, as occurs with any desk-bound job, is associated with increased rates of obesity, impaired cognition, and numerous other chronic diseases (Dunstan et al., 2011; Thyfault et al., 2014; Falck et al., 2016). The majority of adults' weekly waking hours are spent at work, which is invariable sedentary (McCrady and Levine, 2009). Hence, solutions to reverse work-time sitting and encourage daily movement (non-exercise activity thermogenesis [NEAT]) are necessary (Levine, 2010).

Excessive sitting can, in part, be attributed to the computer-based nature of modern work and to the standard office design, both of which encourage employees to remain seated throughout the workday (McCrady and Levine, 2009). Walking or standing while at work are 2 possible solutions for disrupting total workplace sitting time (Dempsey et al., 2016); however, these options are often not practical (Judice et al., 2015; Levin and Chisholm, 2016) because leaving a workstation or office can hinder workflow (Stengard et al., 2016). New methods are needed to help sedentary workers move more.

One approach to decreasing workplace sitting is to transform sitting into an active behavior, termed *dynamic sitting*. Laboratory studies have shown that people who fidget (move) while sitting increase energy expenditure by up to 10% more than those who do not (Levine et al., 2000). In one example of dynamic sitting, office chairs are replaced with large rubber balls (exercise stability ball) (Marks et al., 2012) so that a worker has to continuously fine-tune his or her balance and trunk musculature to maintain posture. Another dynamic sitting solution, such as with the apparatus we tested, is to encourage fidgeting and/or leg movements while seated (Pynt, 2015).

Walking, even slowly, doubles energy expenditure (Bouten et al., 1996; Westerterp et al., 1996); however, sitting, in general, is not exothermic (0–10% increase above basal metabolic rate) (Bouten et al., 1996; Westerterp et al., 1996). Here, we examine whether a commercial apparatus that promotes dynamic sitting can increase energy expenditure and heart rate above resting values. We compared these values to low-speed walking, which is known to improve overall health (Buckley et al., 2015). We hypothesized that the under-the-table dynamic-sitting apparatus we tested was associated with increased energy expenditure compared to sitting in a standard office chair. Because exercise is associated with increased heart rate, which in turn is linked to decreased morbidity and mortality (Chave et al., 1978; Pratley et al., 2000), we assessed the impact of the under-the-table dynamic-sitting apparatus on heart rate as well.

## Participants and methods

### Participants

Participants provided informed written consent and the Mayo Clinic Institutional Review Board approved the protocol. Twenty-six participants (14 women and 12 men) were included with a mean (±SD) age of 23 ± 5 years and a body mass index (BMI) of 26 ± 5.5 kg/m^2^.

### Standard office chair

The criterion model chair (the “control chair”) used is a standard office chair (Steelcase, Grand Rapids, MI).

### Under-the-table leg-movement apparatus

**Figure 1:**
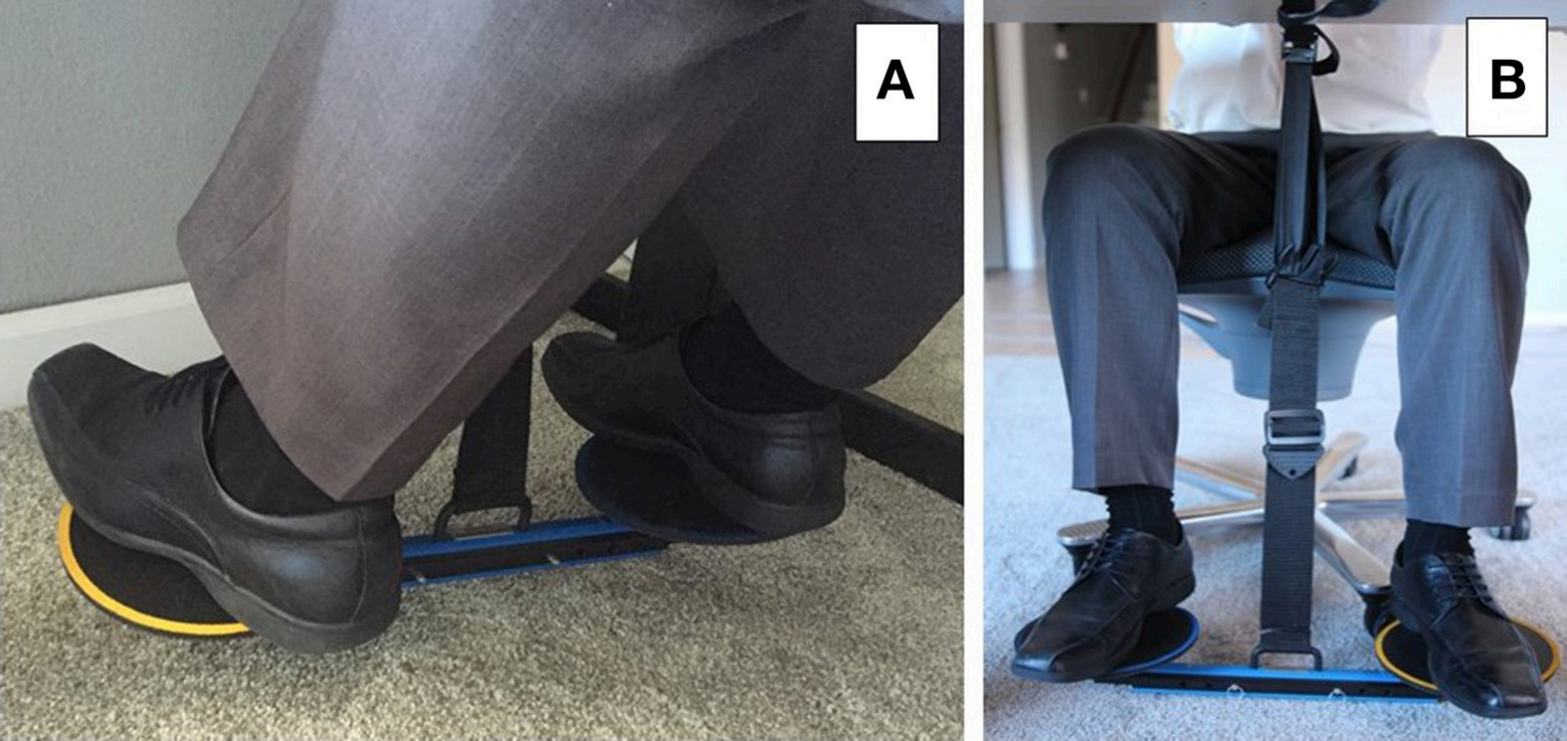
**Under-the-table leg-movement apparatus (A,B)**.

The HOVR (Active Ideas LLC, Chicago, IL) is a pendulum attached to the underside of a desk or a portable stand. At the end of the pendulum are two discs mounted on an adjustable balanced beam (Figure 1).

#### Attachment to the pre-existing desk

At the top of the pendulum is a dense plastic clip. The clip is hung from a metal hook on the bottom side of the desk or 40-cm portable stand designed to fit under a standard office desk. The fastener mounted to the underside of the desk is securely attached with 4 screws. The pendulum may be moved up and down, forward and backward to achieve the user's desired position for both attachment options. In this study, the under-the-desk mount was used.

The pendulum is constructed from a 5-cm-wide nylon webbed strap and is adjustable from approximately 20 to 70 cm. At the bottom of the pendulum is another dense plastic clip, identical to one at the top. This clip is fastened to a metal pin (3.5-cm long and 0.5-cm in diameter). The metal pin is the fulcrum for the adjustable balanced beam and discs (Figure 1).

The balanced beam is constructed from dense plastic and rubber. It is adjustable to 37, 42, and 47 cm with 2 screws to accommodate users' varying sizes and preferences. At each end of the balance beam, identical 16-cm diameter metal discs are mounted with “ball and socket” metal hardware that allows for approximately 20° of motion in any direction relative to the position of the balance beam. The edge (circumference) and the top of the disc are covered by rubber to add a greater friction coefficient, which prevents the participant's feet from slipping. The discs also spin freely on the z-axis to allow leg movement without needing to readjust the feet (Figure 1).

### Protocol

Prior to testing, participants were shown the equipment and the experimental protocol was explained. Body composition, height, weight, and blood pressure were all measured. Participants confirmed that they had not consumed any food or beverage aside from water in the 2 h preceding testing. Patients then rested, sitting comfortably at rest in a shaded quiet room for 30 min. They were not permitted to speak, eat, or use mobile devices during testing.

Participants were tested in thermal comfort (25.2 ± 0.7°C, 956.9 + 1.8 mBar barometric pressure, and 57.0 + 2.1% humidity).

Sitting energy expenditure and heart rate were measured for 20 min via indirect calorimetry. During this time, participants worked at a computer and sat on a standard office chair (Criterion; Steelcase, Grand Rapids, MI) in an effort to simulate their normal work activity. Data for the first 2 and final 2 min were excluded. Following this, subjects rested for 20 min while sitting (not working).

For the next timed interval, participants used the under-the-table dynamic-sitting apparatus while working at the computer. As with the previous segment, energy expenditure and heart rate were measured for 20 min, and data for the first 2 and final 2 min were excluded. Participants again rested in a sitting position for 20 min following testing.

Finally, participants were asked to walk on a calibrated treadmill (4Front; Woodway, Waukesha, WI) at 1 mph and 2 mph, each for 20 min. These speeds were thought to be comparable to the rates of people walking while at work (Ben-Ner et al., 2014). Energy expenditure and heart rate were measured throughout the walks, and data for the first 2 and final 2 min of each velocity were excluded.

The order of the sitting and walking phases were not randomized. This was to avoid the effect of high energy expenditure (as occurs after walking) on lower exertion measurements (e.g., fidgeting). This approach was used when measuring small changes in energy expenditure (Levine et al., 1999, 2000).

## Methods

### Body composition

Participants' body composition and weight were measured using a calibrated Seca Medical Body Composition Analyzer 514 (Seca, Hamburg, Germany) (Heymsfield et al., 2000) while they were wearing light clothing (athletic shorts and t-shirt); height (without shoes) was measured using a Seca 217 stadiometer (Seca, Hamburg, Germany).

### Energy expenditure

Energy expenditure was measured using indirect calorimetry (Metamax 3B; Cortex, Leipzig, Germany) (Levine et al., 2000). The calorimeter was calibrated using 5.0% CO_2_ 15.0% O_2_ balance nitrogen (Praxair Inc., Danbury, CT) and ambient air according to the manufacturer's specifications. In addition, the calorimeter was volume calibrated before each participant using a 3 L syringe. The calorimeter was able to collect breath-by-breath CO_2_ and O_2_ production and consumption, respectively, and energy expenditure was calculated using standard formulae (Weir, 1949).

### Heart rate monitoring

Participants were also fitted with a Polar Heart Rate Monitor H7 (Polar Inc., Lake Success, NY). Heart rate samples were synchronized and recorded for each breath.

### Statistical analysis

Analysis of data with repeated measures needs to consider the covariance structure due to correlations between repeated measures across time or different conditions on each participant. Failure to properly take care of this issue could result in biased estimates. The univariate analysis of variance (ANOVA) assumes equal variances or correlations across time or conditions on each participant, and this might not be true. In many cases, participant correlations tend to decrease with increasing lag time between measures. To overcome this limitation of univariate ANOVA, the general linear mixed model is used in this manuscript. This model allows for different correlations between measures.

For analysis, the PROC MIXED with REPEATED statement was used in SAS (SAS Institute Inc., Cary, NC). The model assumed no specific variance-covariance structure (unstructured) based on Akaike Information Criterion values and –2 log likelihood scores of 4 models (unstructured, compound symmetry, auto-regressive, and auto-regressive heterogeneous variance-covariance). The original data in wide format consisted of 26 participants (14 women, 12 men), and 2 outcomes (energy expenditure and heart rate) were measured under 5 different conditions for each participant. The data were transposed to a long format for the linear mixed model, and total available sample size for analysis was 130 person-conditions (26 individuals × 5 conditions).

## Results

Participants tolerated the protocol without complaint. Anthropometric and body composition data are shown in Table 1. Four additional participants were studied (3 women, 1 man), but their data are not included in the analysis because it was incomplete due to technical failures. Omitting these 4 subjects did not influence the principal conclusion because, in all 4 cases, energy expenditure increased using the under-the-table dynamic-sitting apparatus.

**Table 1:** **Demographic and body composition information for 26 study volunteers^a^**.

	**Women**				**Men**				**Total**			
	**Mean**	***SD***	**Min**	**Max**	**Mean**	***SD***	**Min**	**Max**	**Mean**	***SD***	**Min**	**Max**
Height (cm)	165.2	3.7	156.0	170.7	176.9	5.7	167.4	187.5	170.6	7.5	156.0	187.5
Weight (kg)	71.7	20.4	46.4	118.1	81.5	15.4	66.8	121.3	76.2	18.6	46.4	121.3
BMI (kg/m^2^)	26.4	8.0	16.6	42.3	26.0	4.6	20.9	37.3	26.2	6.5	16.6	42.3
Age (years)	38.2	16.7	19.0	64.0	26.7	8.5	18.0	44.0	32.9	14.5	18.0	64.0
BP: Systolic	116.3	18.8	94.0	157.0	114.6	14.2	93.0	145.0	115.5	16.6	93.0	157.0
BP: Diastolic	76.2	11.4	63.0	104.0	75.8	10.9	56.0	98.0	76.0	11.0	56.0	104.0
Body fat (%)	38.1	19.3	16.0	94.5	20.0	9.4	9.0	38.0	29.8	17.8	9.0	94.5
No. of patients	14				12				26			

a *Body fat was measured using bioelectrical impedance (Falck et al., 2016)*.

*BMI, body mass index; BP, blood pressure; SD, standard deviation*.

Twenty-three of the participants reported that their jobs were sedentary in nature, whereas the remaining 3 reported having employment that necessitated a degree of movement throughout the workday. Of the 23 participants, 7 self-reported as being sedentary, 12 as being moderately active, and 6 as exercising regularly.

Energy expenditure for the 2 seated conditions (standard chair and under-the-table dynamic-sitting apparatus) and slow walking (1 and 2 mph) are shown in Tables 2, 3. Energy expenditure while sitting in a standard chair showed a positive correlation with body weight (*r* = 0.55, *P* = 0.003). The relationship was described by the following equation:

**Table 2:** **Energy expenditure and heart rate by sex**.

**Phase**	**Energy expenditure (kcal per h)**			**Heart rate (bpm)**		
	**Women**	**Men**	**Total**	**Women**	**Men**	**Whole group**
	**Mean ± *SD***	**Mean ± *SD***	**Mean ± *SD***	**Mean ± *SD***	**Mean ± *SD***	**Mean ± *SD***
Sitting	69.7 ± 12.8	94.5 ± 14.2	81.2 ± 18.2	71.2 ± 8.6	75.7 ± 15.9	73.2 ± 12.4
Using apparatus	81.7 ± 17.9^a^	112.1 ± 116.1^a^	95.7 ± 22.8^a^	73.4 ± 9.0^a^	77.6 ± 17.6	75.3 ± 13.6^a^
Walking at 1 mph	150.8 ± 43.8^a^^,^ ^b^	187.0 ± 42.5^a^^,^ ^b^	167.5 ± 46.1^a^^,^ ^b^	87.2 ± 13.4^a^^,^ ^b^	94.0 ± 41.4^a^^,^ ^b^	90.3 ± 29.3^a^^,^ ^b^
Walking at 2 mph	186.8 ± 55.4^a^^,^ ^b^^,^ ^c^	225.9 ± 35.8^a^^,^ ^b^^,^ ^c^	204.9 ± 50.5^a^^,^ ^b^^,^ ^c^	88.7 ± 13.4^a^^,^ ^b^	101.3 ± 51.2^a^^,^ ^b^	94.5 ± 35.9^a^^,^ ^b^^,^ ^c^
Walking at 3 mph	261.4 ± 87.6^a^^,^ ^b^^,^ ^c^^,^ ^d^	294.3 ± 48.6^a^^,^ ^b^^,^ ^c^^,^ ^d^	276.6 ± 72.8^a^^,^ ^b^^,^ ^c^^,^ ^d^	106.1 ± 17.7^a^^,^ ^b^^,^ ^c^^,^ ^d^	115.7 ± 73.6^a^^,^ ^b^^,^ ^c^^,^ ^d^	110.6 ± 50.7^a^^,^ ^b^^,^ ^c^^,^ ^d^

a *Significantly different from “sitting” condition at the P-value 0.05 level*.

b *Significantly different from “surfing” condition at the P-value 0.05 level*.

c *Significantly different from “1 mph” condition at the P-value 0.05 level*.

d *Significantly different from “2 mph” condition at the P-value 0.05 level. “Apparatus” refers to the apparatus to promote leg movement. SD, standard deviation*.

**Table 3:** **Energy expenditure and heart rate by weight and sex**.

	**Energy Expenditure (kcal/h/kg)**		
	**Women**	**Men**	**Total**
**Phase**	**Mean ± *SD***	**Mean ± *SD***	**Mean ± *SD***
Sitting	1.0 ± 0.2	1.2 ± 0.2	1.1 ± 0.2
Using apparatus	1.2 ± 0.2^a^	1.4 ± 0.2^a^	1.3 ± 0.2^a^
1 mph	2.1 ± 0.3^a^^,^ ^b^	2.3 ± 0.3^a^^,^ ^b^	2.2 ± 0.3^a^^,^ ^b^
2 mph	2.6 ± 0.3^a^^,^ ^b^^,^ ^c^	2.8 ± 0.2^a^^,^ ^b^^,^ ^c^	2.7 ± 0.3^a^^,^ ^b^^,^ ^c^
3 mph	3.6 ± 0.3^a^^,^ ^b^^,^ ^c^^,^ ^d^	3.6 ± 0.3^a^^,^ ^b^^,^ ^c^^,^ ^d^	3.6 ± 0.3^a^^,^ ^b^^,^ ^c^^,^ ^d^

a *significantly different from “sitting” condition at the P-value 0.05 level*.

b *Significantly different from “surfing” condition at the P-value 0.05 level*.

c *Significantly different from “1 mph” condition at the P-value 0.05 level*.

d *Significantly different from “2 mph” condition at the P-value 0.05 level. “Apparatus” refers to the apparatus to promote leg movement. SD, standard deviation*.

Sitting energy expenditure (kcal/hr) = 0.544 × weight (kg) + 39.7.

**Figure 2:**
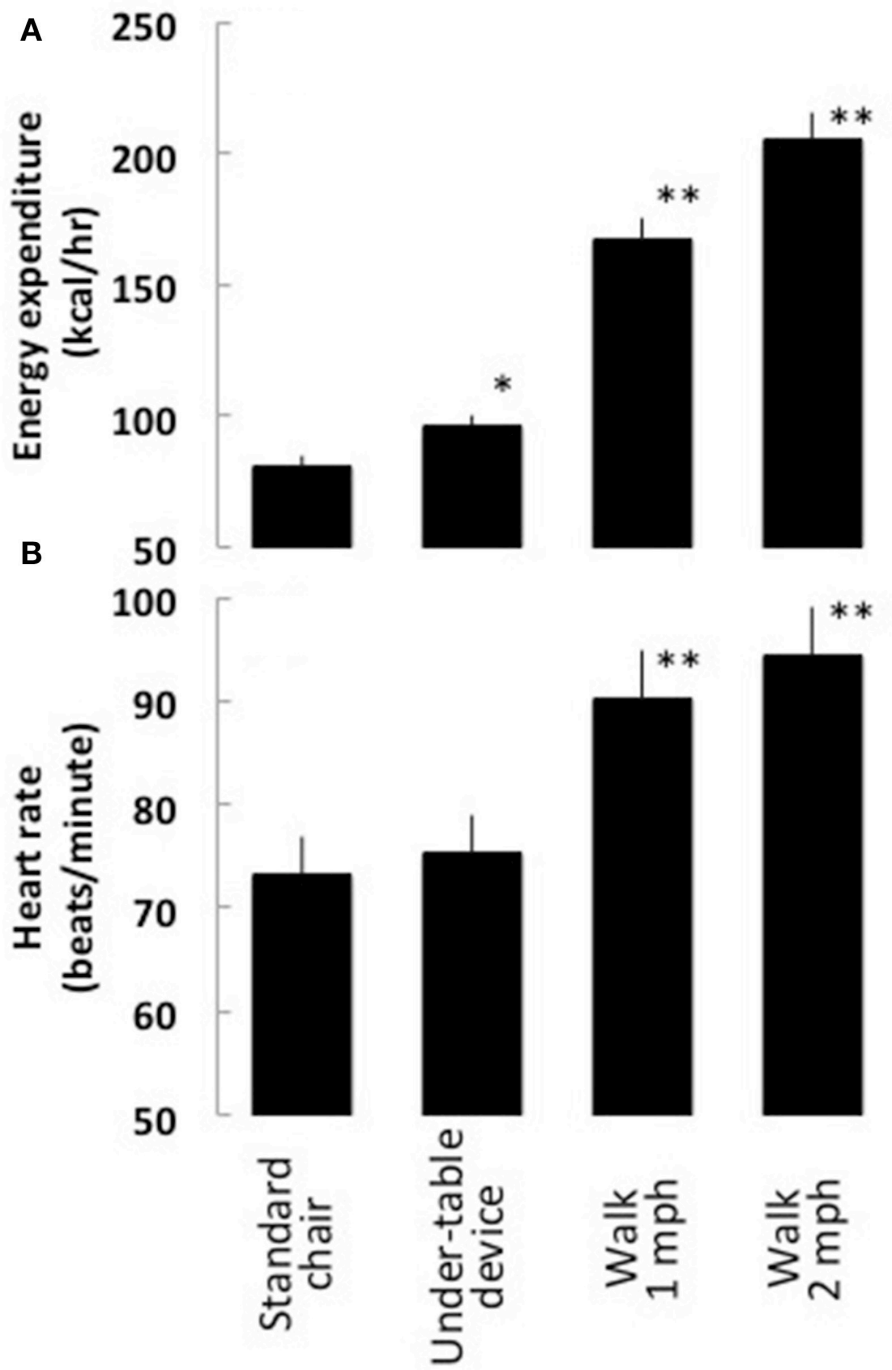
**Energy expenditure and heart rate**.

Energy expenditure increased considerably while using the under-the-table dynamic-sitting apparatus when compared to a standard office chair (Tables 2, 3). Energy expenditure increased in 25 of 26 participants, from a mean of 81 ± 18 kcal/hr to 96 ± 23 kcal/h (*P* < 0.001), representing a mean increase of 18.4 ± 16.2%. There was a strong association between energy expenditure while sitting on a standard chair and energy expenditure using the under-the-table leg-movement apparatus (*r*^2^ = 0.76; *P* < 0.001). Heart rate did not increase substantially when using the under-the-table leg-movement apparatus compared to sitting on a standard office chair without the apparatus (73 ± 12 cf 75 ± 14 beats/min) (Figure 2).

Changes in energy expenditure for the under-the-table leg-movement apparatus vs. the standard office chair were not as great as for walking at a speed of 1 or 2 mph (Tables 2, 3). The changes in energy expenditure were 15 ± 11 kcal/hr for the under-the-table leg-movement apparatus, 86 ± 24 kcal/hr for walking at 1 mph, and 124 ± 39 kcal/hr for walking at 2 mph. Slow walking at 1 and 2 mph were associated with significant increases in heart rate (rest, 73 ± 12 bpm; 1 mph, 90 ± 36 bpm; [*P* < 0.001]; and 2 mph, 111 ± 51 bpm [*P* < 0.001]) when compared to sitting in a standard office chair.

The results show that there is a significant difference in the overall level of energy expenditure between men and women (*P* = 0.04). However, these differences disappear after body weight is accounted for (Table 3). There were no differences in the overall heart rate level between men and women. However, there is a considerable conditioning effect whereby heart rate increased with walking, as was expected.

## Discussion

Excessive sitting is linked with chronic disease, impaired cognition, and obesity (Dunstan et al., 2011; Thyfault et al., 2014; Falck et al., 2016). The majority of adults' weekly waking hours are spent at work, which is invariable sedentary (McCrady and Levine, 2009). Hence, solutions to considerably decrease work-time sitting and encourage daily movement are necessary. In this study, we found that when a person sat and used an under-the-table dynamic-sitting apparatus, energy expenditure increased by about 20%. Heart rate, however, did not increase substantially. The reason for this is that the movement promoted by the under-the-table apparatus is sufficient to increase energy expenditure through leg muscle activity, but not sufficiently intense enough to accelerate heart rate markedly (Levine et al., 2000). It is not surprising that energy expenditure increased significantly based on leg movements alone because gluteal-femoral muscular contractions contribute substantially to human energy expenditure (Westerterp et al., 1996; Westerterp and Bouten, 1997). What is important to note is that these types of movements may directly impact glycemic control and other health outcomes (Kadam and Chuan, 2016; Dempsey et al., 2017; Fanchamps et al., 2017; Larsen et al., 2017) although we did not measure these outcomes. The under-the-table dynamic-sitting apparatus we tested was exothermic but unlikely to contribute to aerobic fitness. Noting that heart rate did not increase with the use of the under-the-table dynamic-sitting apparatus it could be assumed that such a device doesn't contribute to physical fitness. It may not. However, it is possible that by using the under-the-table dynamic-sitting apparatus a person becomes more active throughout their day and daily physical activity increases. However, this was not tested here. Other studies show that office furniture, such as treadmill desks, can promote NEAT and daily activity (Koepp et al., 2013; Ben-Ner et al., 2014). These approaches, while expensive, have improved health care outcomes and workplace productivity (Koepp et al., 2013; Ben-Ner et al., 2014). Active work has the potential to improve overall health.

## Limitations

Our study had several limitations. As this was a laboratory study conducted only to examine the effects of an apparatus on energy expenditure and heart rate, we did not examine whether the apparatus would impact productivity (positively or negatively), health outcomes, or standing time; these would be goals of future studies. There is solid evidence that breaking up sitting time can benefit glycemic variables (Dunstan et al., 2012). We did not examine whether the apparatus we studied could benefit blood glucose; this too would be a beneficial future study. Similarly, more time spent walking is known to improve overall health (Levine, 2007). It would be interesting to assess whether using a dynamic-sitting apparatus could help increase daily walking. In spite of these limitations, these experiments are encouraging. It would be worthwhile to examine dynamic-sitting interventions in real-world offices.

## Conclusions

In conclusion, new approaches are needed to help decrease excessive sitting and the poor health linked with this prolonged lack of physical activity. Here, we have shown that an under-the-table dynamic-sitting apparatus can improve energy expenditure while a person sits. The applicability of such an apparatus in real-world offices remains to be seen.

## Author contributions

GK was responsible for study design, IRB approvals, patient recruitment, data collection, data analysis, and manuscript preparation. JL was responsible for study design, IRB approvals, data analysis, and manuscript preparation. GM was responsible for data collection and data analysis.

## Funding

Funds to complete this study were provided by Mayo Clinic Foundation.

### Conflict of interest statement

The authors declare that the research was conducted in the absence of any commercial or financial relationships that could be construed as a potential conflict of interest.
